# Supplementary material for: Core conserved transcriptional regulatory networks define the invasive trophoblast cell lineage
Source: Development. 2023 Jul 31;150(15):dev201826. doi: 10.1242/dev.201826 (PMC10445752; doi:10.1242/dev.201826)
Supplement: Supplementary information [file develop-150-201826-s1.pdf]

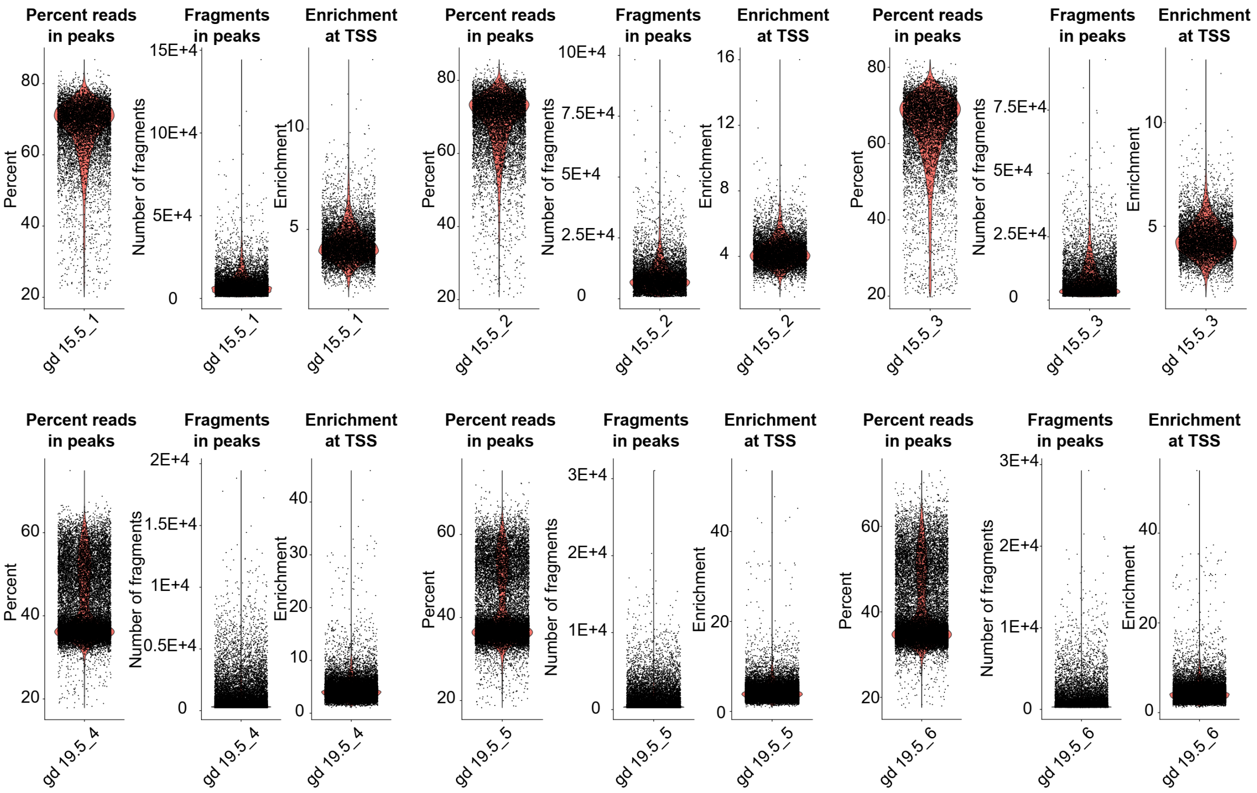

Fig. S1. Quality control of single-nucleus ATAC sequencing (snATAC-seq) data. Violin plots showing distributions of percent of reads in peaks, numbers of fragments in peaks, and chromatin accessibility enrichment at transcription start sites (**TSS**). Nuclei with the percent of reads in peaks >15%, numbers of fragments in peaks in the range from 1000 to 20000, and chromatin accessibility enrichment >1.5 were retained.

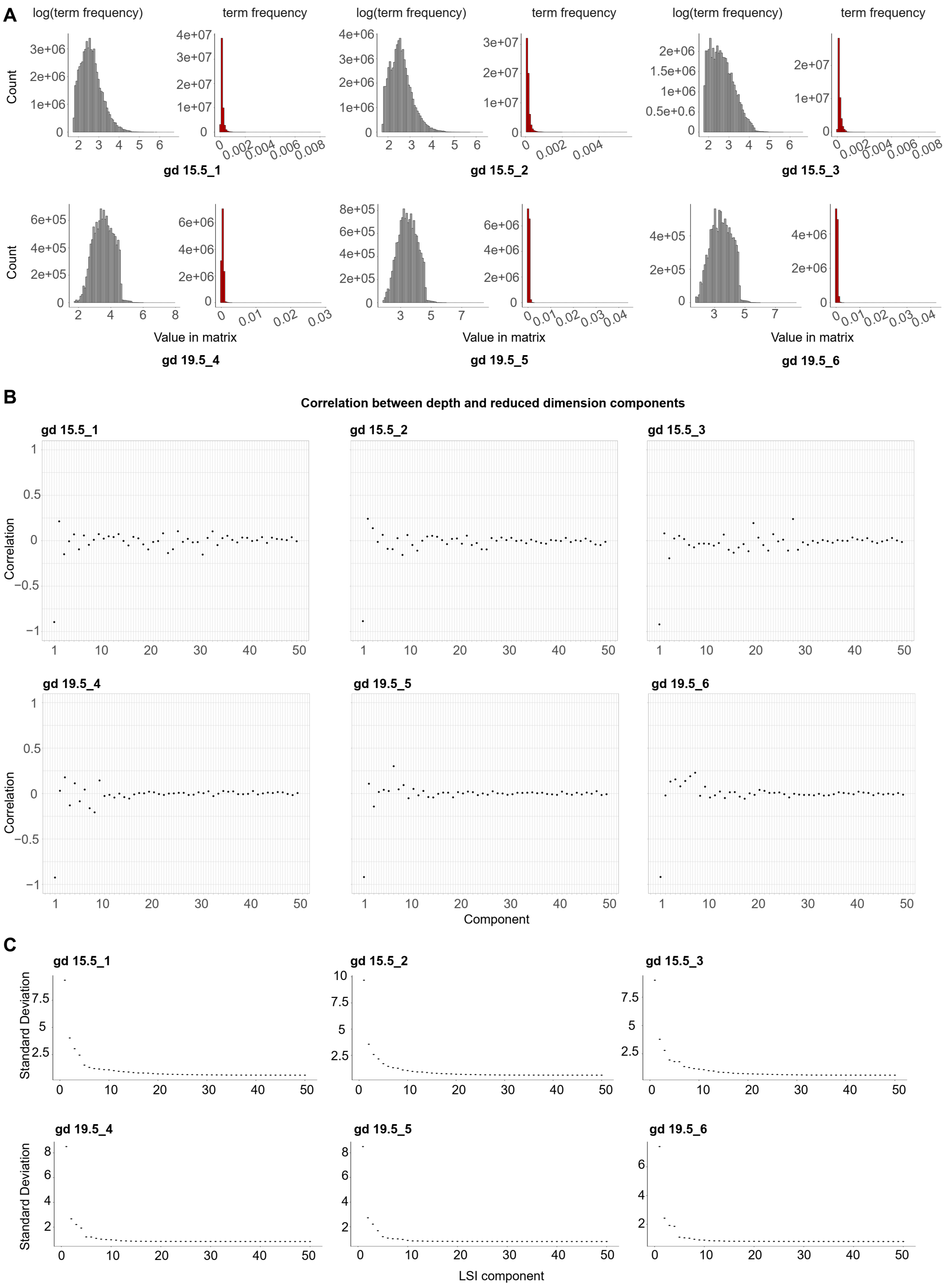
A
log(term frequency)
term frequency
Count
Value in matrix
gd 15.5_1
gd 15.5_2
gd 15.5_3
gd 19.5_4
gd 19.5_5
gd 19.5_6
B
Correlation between depth and reduced dimension components
Correlation
Component
C
Standard Deviation
LSI component

Fig. S2. Processing of single-nucleus ATAC sequencing (snATAC-seq) data. A) Histogram of chromatin accessibility count matrices showing a trend of skewness. As a result, we used method =3 which computes log(term frequency) × log(IDF) for term frequency inverse document frequency normalization. Abbreviations: log(term frequency), method where log(term frequency) × log(IDF) is calculated; term frequency, method where log(term frequency × IDF) is calculated. **B**) Correlation between library depth and reduced dimension components showing that the first component across replicates were highly correlated with library depth. Therefore, the first component was excluded in the analyses. **C**) Elbow plots showing the amount of standard deviation each latent semantic indexing (**LSI**) component represented. The first 20 components of gd 15.5 samples, and 10 components of gd 19.5 samples, captured most of the variation in the data.

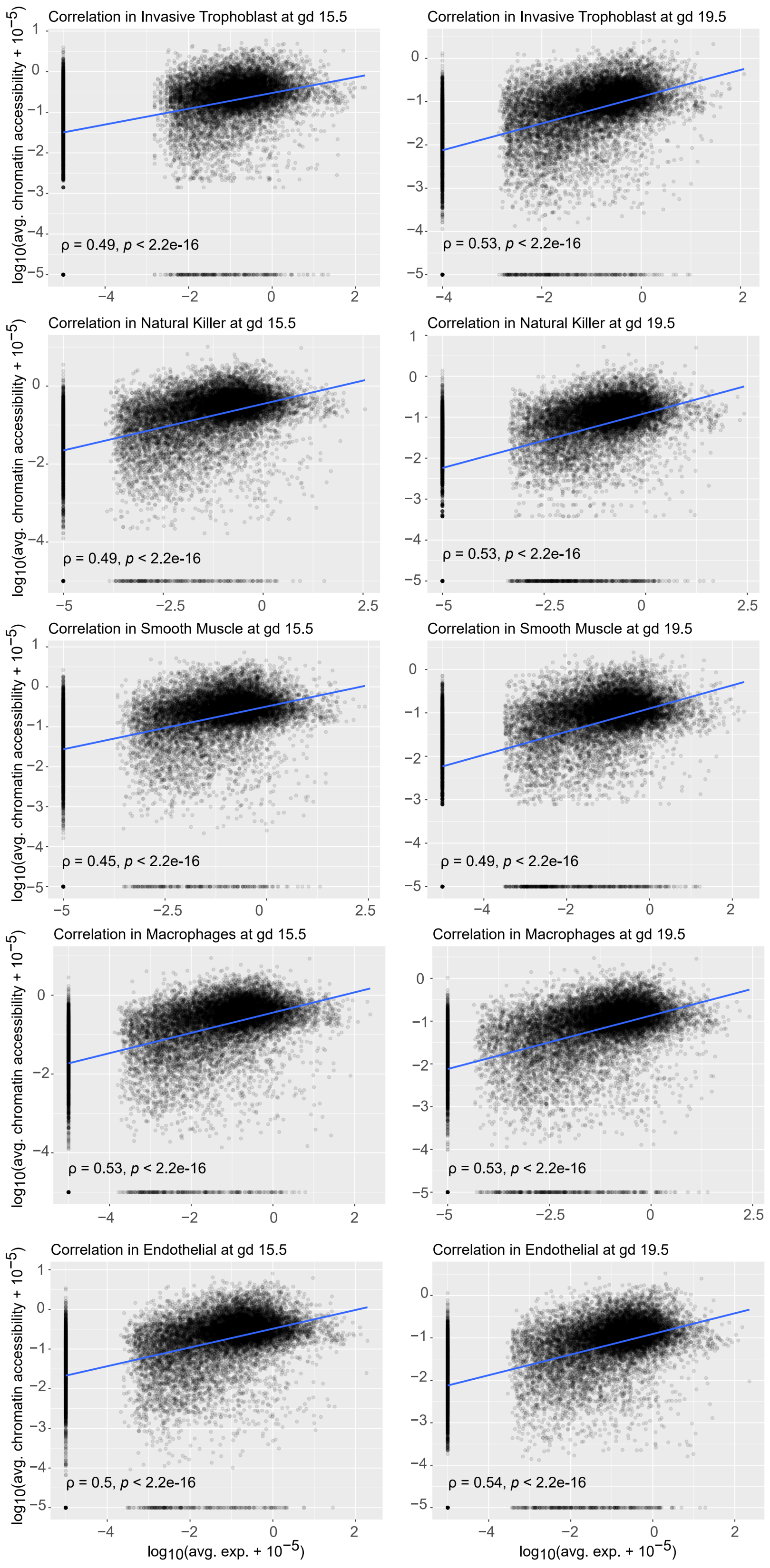
Correlation in Invasive Trophoblast at gd 15.5
ρ = 0.49, p < 2.2e-16
Correlation in Invasive Trophoblast at gd 19.5
ρ = 0.53, p < 2.2e-16
Correlation in Natural Killer at gd 15.5
ρ = 0.49, p < 2.2e-16
Correlation in Natural Killer at gd 19.5
ρ = 0.53, p < 2.2e-16
Correlation in Smooth Muscle at gd 15.5
ρ = 0.45, p < 2.2e-16
Correlation in Smooth Muscle at gd 19.5
ρ = 0.49, p < 2.2e-16
Correlation in Macrophages at gd 15.5
ρ = 0.53, p < 2.2e-16
Correlation in Macrophages at gd 19.5
ρ = 0.53, p < 2.2e-16
Correlation in Endothelial at gd 15.5
ρ = 0.5, p < 2.2e-16
Correlation in Endothelial at gd 19.5
ρ = 0.54, p < 2.2e-16
log10(avg. chromatin accessibility + 10^−5)
log10(avg. exp. + 10^−5)

Fig. S3. Correlation between gene expression and predicted gene activity using chromatin accessibility profiles. Scatter plots showing Spearman correlations between gene expression and predicted gene activity using chromatin accessibility profiles. At both gestation days, gene expression (x-axis) and predicted gene activity (y-axis) were moderately but significantly correlated for each of the cell populations: invasive trophoblast, natural killer, smooth muscle, macrophage and endothelial cells.

Development • Supplementary information

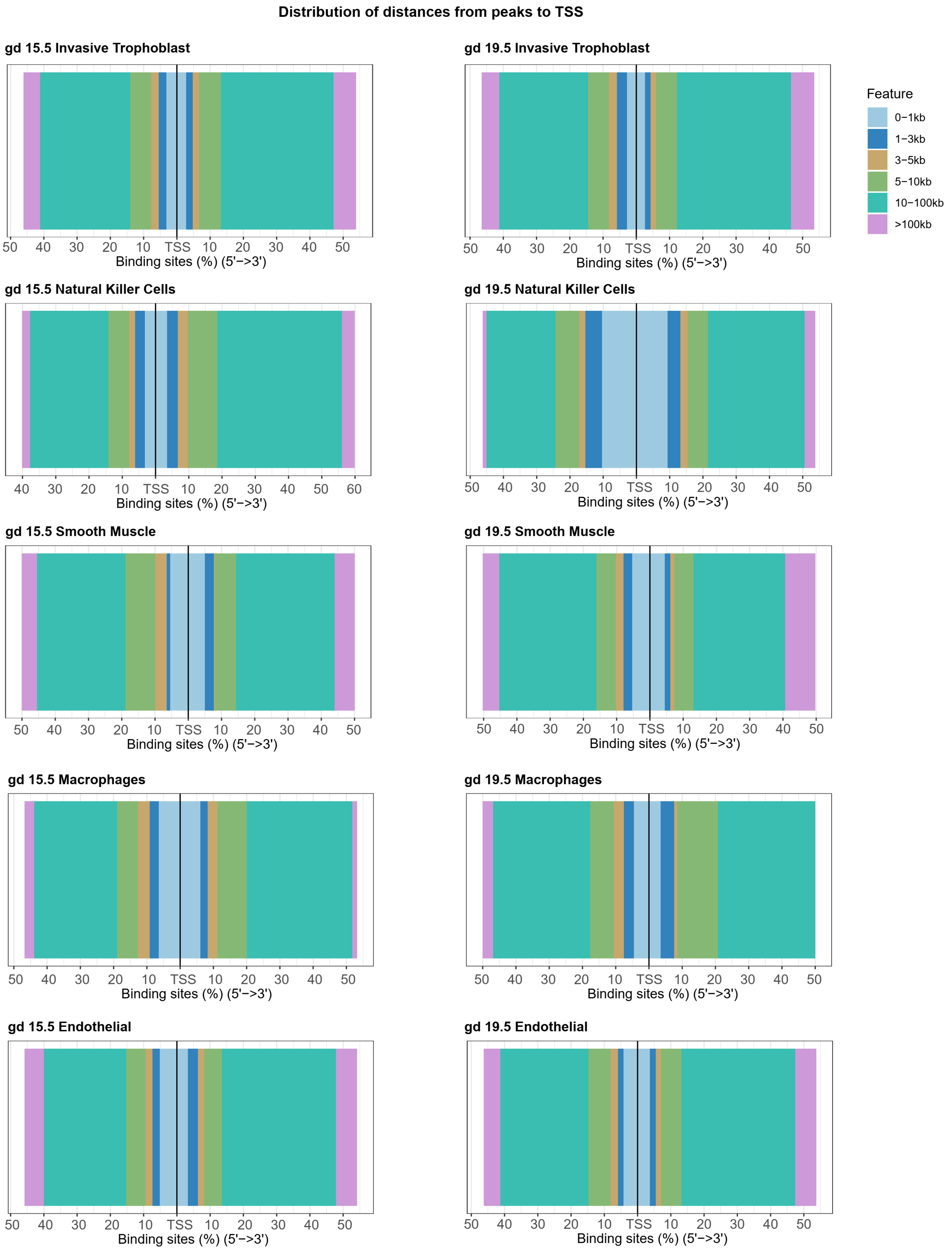

Fig. S4. Distribution of distances between open regions and transcription start sites (TSS). Stack bar plots showing that cell type-specific open chromatin peaks were most frequently distal to the TSS.

Development • Supplementary information

A

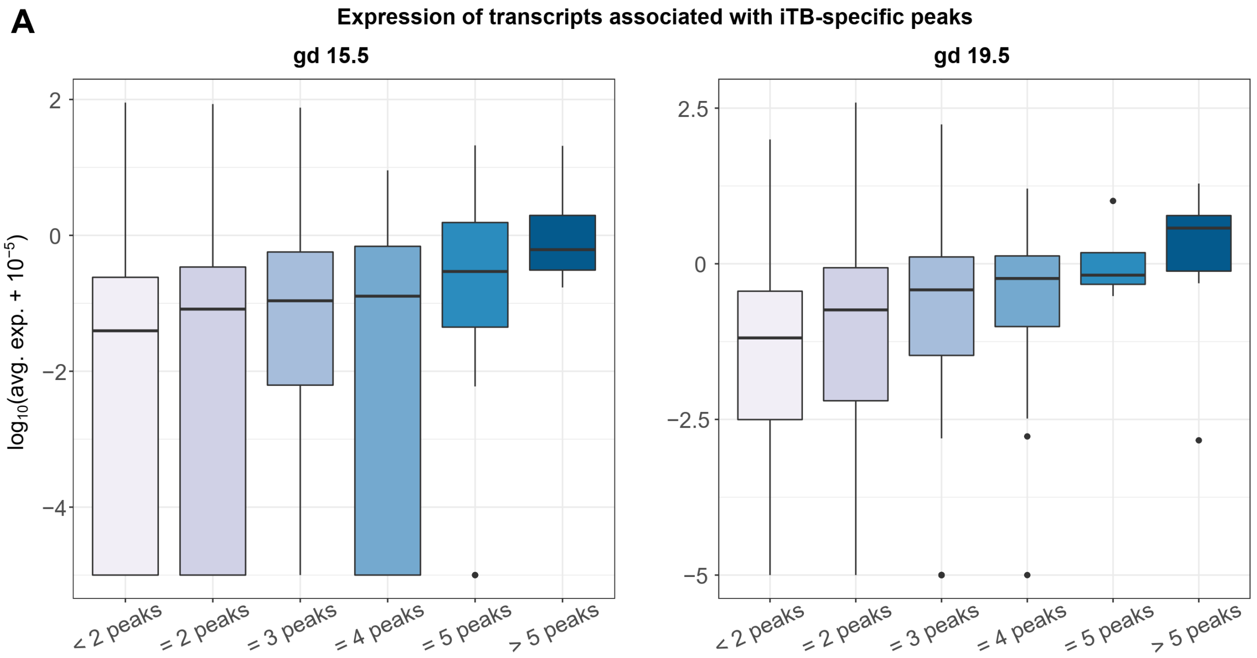

B

	gd 15.5		gd 19.5	
Test	Adjusted P-value	Significance	Adjusted P-value	Significance
"< 2 peaks" vs "≥ 2 peaks"	6.48E-08	*	2.60E-09	*
"= 2 peaks" vs "≥ 3 peaks"	6.00E-04	*	1.35E-02	*
"= 2 and 3 peaks" vs "≥ 4 peaks"	7.60E-05	*	1.42E-02	*
"= 2, 3 and 4 peaks" vs "≥ 5 peaks"	5.70E-07	*	0.018691913	*
"< 3 peaks" vs "≥ 3 peaks"	3.51E-08	*	6.87E-08	*
"= 3 peaks" vs "≥ 4 peaks"	7.06E-03	*	1.10E-01	
"= 3 and 4 peaks" vs "≥ 5 peaks"	3.18E-05	*	0.066567942	
"< 4 peaks" vs "≥ 4 peaks"	2.22E-07	*	1.01E-04	*
"= 4 peaks" vs "≥ 5 peaks"	1.70E-03	*	0.244923802	
"< 5 peaks" vs "≥ 5 peaks"	1.87E-08	*	0.001980376	*

Fig. S5. Analysis of the relationship between gene expression and the number of peaks associated with a gene. A) Boxplots of transcript expression associated with invasive trophoblast (**iTB**) cell-specific peaks. Expression was plotted in a $\log_{10}$(average expression + 10^{-5}) scale. **B**) Adjusted p-values reported when comparing transcript expression profiles in different groups. * indicates the difference is significant. Statistical analyses were performed using Wilcoxon rank sum test at a significance level of 0.05.

Development • Supplementary information

Table S1. Details on the number of nuclei before and after quality control; cell type-specific open regions and their peak to gene associations.

Click here to download Table S1

Table S2. Peak counts per gene in rat invasive trophoblast cell population (**iTB**) at gestational day (**gd**) 15.5 and 19.5 and in human extravillous trophoblast cells (**EVT**); gene expression in rat iTB at gd 15.5 and 19.5 according to scRNA-seq data.

Click here to download Table S2

Table S3. Analysis of common peaks in rat iTB with details of peak coordinates in both rat and human, gene ontology analysis, motif enrichment analysis and peak to gene associations.

Click here to download Table S3

Table S4. Stage-specific open regions in rat iTB populations and peak to gene associations.

Click here to download Table S4

Table S5. Analysis of conserved common peaks in rat iTB with details of conserved peak coordinates, conserved common peaks overlapping with H3k27ac ChIP-seq peaks in EVT, motif enrichment analysis, network construction and network metrics.

Click here to download Table S5
